# dsSynthetic: synthetic data generation for the DataSHIELD federated analysis system

**DOI:** 10.1186/s13104-022-06111-2

**Published:** 2022-06-27

**Authors:** Soumya Banerjee, Tom R. P. Bishop

**Affiliations:** Medical Research Council Epidemiology Unit, University of Cambridge School of Clinical Medicine, Cambridge, UK

**Keywords:** Synthetic data, Data harmonisation, Federated analysis

## Abstract

****Objective**:**

Platforms such as DataSHIELD allow users to analyse sensitive data remotely, without having full access to the detailed data items (federated analysis). While this feature helps to overcome difficulties with data sharing, it can make it challenging to write code without full visibility of the data. One solution is to generate realistic, non-disclosive synthetic data that can be transferred to the analyst so they can perfect their code without the access limitation. When this process is complete, they can run the code on the real data.

****Results**:**

We have created a package in DataSHIELD (dsSynthetic) which allows generation of realistic synthetic data, building on existing packages. In our paper and accompanying tutorial we demonstrate how the use of synthetic data generated with our package can help DataSHIELD users with tasks such as writing analysis scripts and harmonising data to common scales and measures.

## Introduction

Research is increasingly dependent on the analysis and interpretation of sensitive, personal data (or data that cannot be easily shared) or on the co-analysis of such data from several sources simultaneously. Making data available so that it can be used, may be impeded by ethico-legal processes or by fear of loss of control of data. This results in a lot of work to get permission to gather several datasets together in a central location.

DataSHIELD provides a novel solution that can circumvent some of these challenges [1, 2]. The key feature of DataSHIELD is that data stay on a server at each of the institutions that are responsible for the data. Each institution has control over who can access their data. The platform allows a user to pass commands to each server and the analyst receives results that are designed to only disclose the summary data. For example, the user can fit a linear model to the data but not see the residuals. Furthermore, DataSHIELD enforces other disclosure protection requirements such as requiring a minimum cell count of data points in a summary [3]. Thus DataSHIELD can be used to analyse data without physically sharing it with the users. We refer to this as federated analysis.

A challenge of this approach is that it is harder for data analysts to write and debug code that uses the data when the data are not tangibly in front of them. This is because DataSHIELD prevents access to the data at a detailed level. It is similar to trying to build a Lego model while blindfolded. While it is possible for the user to understand what is happening by asking for summary statistics, this process can still be difficult. For example, to confirm that a subset operation into male and female groups has been successful, the analyst could ask for a summary of the original gender column and check whether the counts of male and female participants match the length of the subset objects. With the datasets in front of them it is immediately obvious if a problem has occurred.

### Hypothesis for using synthetic data

R packages like *synthpop* [4] have been developed to generate realistic synthetic data that is not disclosive: the synthetic data is representative of the real data, but is less likely to enable recovery of the real data. *synthpop* uses classification and regression trees (CART) [5] to generate synthetic data. *synthpop* fits a sequence of regression models. Once the models are fit, synthetic values are generated by drawing from the predictive distributions.

In DataSHIELD, the user (“client”) passes analytical commands to multiple datasets held on remote servers. If the user could have a client side synthetic copy of the real data that is held on each of the servers they are using, this could make code writing and debugging easier. They could have full access to the synthetic data, and confirm that their code is working correctly before applying it to the real data held on the servers.

The user therefore has the benefit of being able to see the data they are working with, but without the need to go through laborious data transfer processes.

Other packages that provide synthetic data generation are *simstudy* [6] and *gcipdr* [7]. *simstudy* [6] requires the user to define the characteristics of variables and their relationships. Non-disclosive access via DataSHIELD can provide these summary statistics. Likewise, *gcipdr* [7] requires users to extract features such as mean, standard deviation and correlations via DataSHIELD, and uses these to provide automated generation of the synthetic data.

We introduce a package (*dsSynthetic*) which provides functionality for generating a local, non-disclosive synthetic copy of data held on remote servers via DataSHIELD. We build on the *synthpop* [4] and *simstudy* packages [6].

Our objective is to generate synthetic data to allow users to more easily design, implement and test code. This code can then be applied on the real data.

## Main text

### Overview of steps for generating synthetic data

We have written the dsSynthetic and partner dsSyntheticClient R packages (hereafter dsSynthetic) to help users to write code for data that is available in a DataSHIELD setting. A tutorial covering the examples in this text with executable code is available here:

https://tombisho.github.io/synthetic_bookdown

We describe the steps for generating synthetic data below:

1. The data custodian uploads the raw data to the server side and installs the server side package *dsSynthetic*.
2. The user installs the packages *dsBaseClient* and *dsSyntheticClient* on the client side.
3. The user calls functions in the *dsSyntheticClient* package to generate a synthetic but non-disclosive data set which is transferred from the server to the client side. The generation of the synthetic data can use methods from: a) synthpop, where the synthetic data are generated on the server side and returned to the client, or b) simstudy where non-disclosive summary characteristics of and relationships between variables are generated on the server side, returned to the client, and the synthetic data are generated on the client side.
4. With the synthetic data on the client side, the user can view the data and write code. They will be able to see the results of the code for the whole synthetic dataset.
5. When the code is complete, it can be implemented on the server using the real data.

**Fig. 1:**
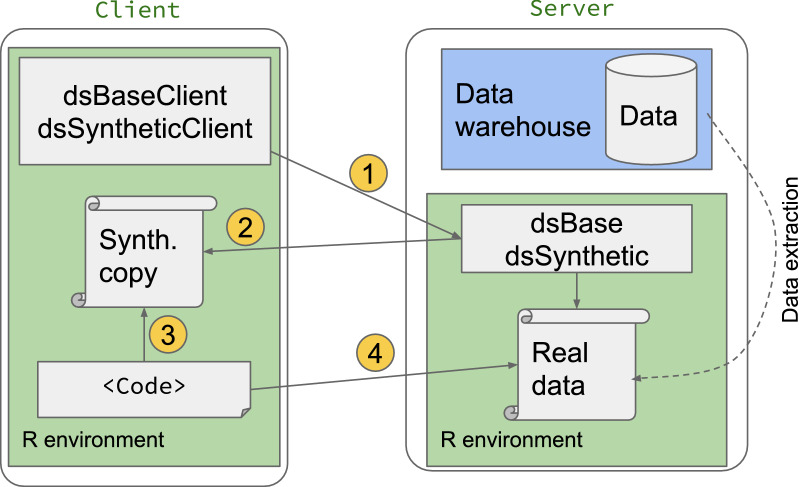
A schematic of generating synthetic data with dsSynthetic, and using it to write, debug and test code. The final code is then used on real data on the server 1. User requests synthetic copy of real data 2. Synthetic copy of data generated on server side and passed to client 3. User writes code against synthetic data on client side 4. Code run on server side with real data

These variations are shown in Fig. 1.

The computational steps are outlined below.

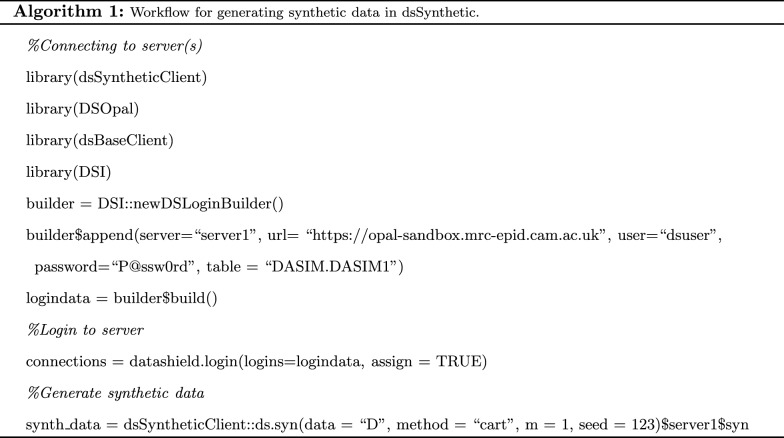



### Using synthetic data to build a DataSHIELD analysis script

**Fig. 2:**
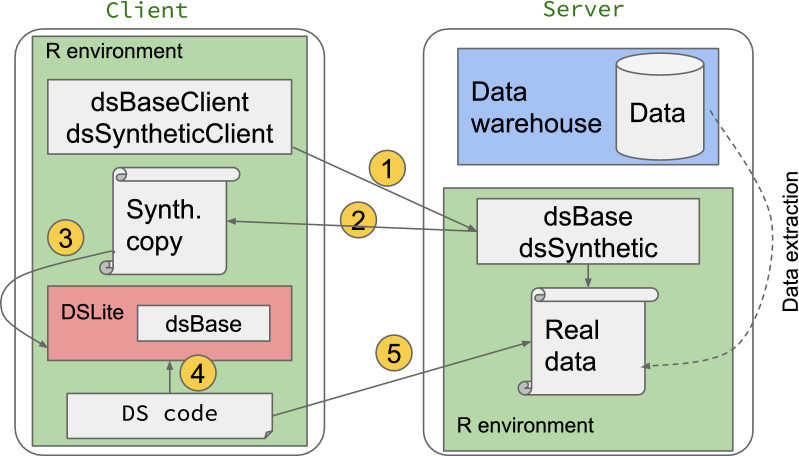
Writing an analysis script via synthetic data without full access. The steps are the following: 1. User requests synthetic copy of real data 2. Synthetic data generated on server side and passed to client 3. Synthetic data held in DSLite environment that simulates server side DataSHIELD environment 4. Develop code against synthetic data in DSLite (with full access) 5. Run code against real data

A key use-case for *dsSynthetic* is to aid analysts in writing DataSHIELD analysis scripts in the standard DataSHIELD scenario where the real data cannot be fully accessed. We assume that the data sets are ready for analysis at different sites, the *dsSynthetic* package is installed on the servers and the *dsSyntheticClient* package is installed on the analyst’s computer (client). This use-case is demonstrated in Fig. 2.

1. Generate the synthetic data so that it is available on the client (this could be data from multiple servers).
2. Load the data into *DSLite* [8]. To test our DataSHIELD script locally on the client, we need to replicate the server environment(s) (which only accepts DataSHIELD function input) on the client. This is provided by the *DSLite* package. A DSLite instance exists in a user’s local R session and behaves the same as a server, except that it allows full access to the data held on it. Therefore the script can be developed against the DSLite instance and at any time the user can view what is happening on DSLite. This makes it easier for the user to correct any problems.
3. Once finished, the script is run on real data on the remote server(s). This step should now run smoothly as any problems with the code have been identified when working with the synthetic data.

### Using synthetic data to write harmonisation code

Prior to analysis with DataSHIELD, data must be harmonised so that variables have the same definition across datasets from all sites [9]. Common solutions to achieving harmonisation are:

1. Have each data custodian harmonise their data and host it on the server. This requires a lot of coordination across groups and there can be a lack of visibility of how harmonisation was achieved.
2. Make a one-off transfer to a central group which does the harmonising work before the data are returned to the custodian for the analysis phase by multiple parties. This suffers the same challenges of a data transfer for analysis.

To avoid these challenges a third way is for a central group to write harmonisation code in Opal [10] which can be used as the hosting datawarehouse on each of the participating servers. This code acts on the raw data on each server to generate datasets which contain harmonised variables. That is, all the variables are on common scales and measures (e.g. all measures of height in metres). To eliminate the need for a data transfer or full data access agreement, the users log into Opal to write the harmonisation code. This means they may not have full access to the data, only to summary statistics.

Again, this makes it challenging to write and test the code. A further complication is that Opal requires the code to be written in MagmaScript, which is based on JavaScript. This language is generally unfamiliar to users that do not have a background in software development. dsSynthetic allows users to write and test MagmaScript on a local synthetic copy of data, before implementing it on the server running Opal.

As with the analysis use case, this testing phase is performed in the R environment in DataSHIELD, and implemented on the server once the code is working properly. Our package enables this by generating synthetic data. We then use the V8 package [11] to run MagmaScript and JavaScript within R. This tested MagmaScript code can then be pasted into the Opal server to run against the real data.

**Fig. 3:**
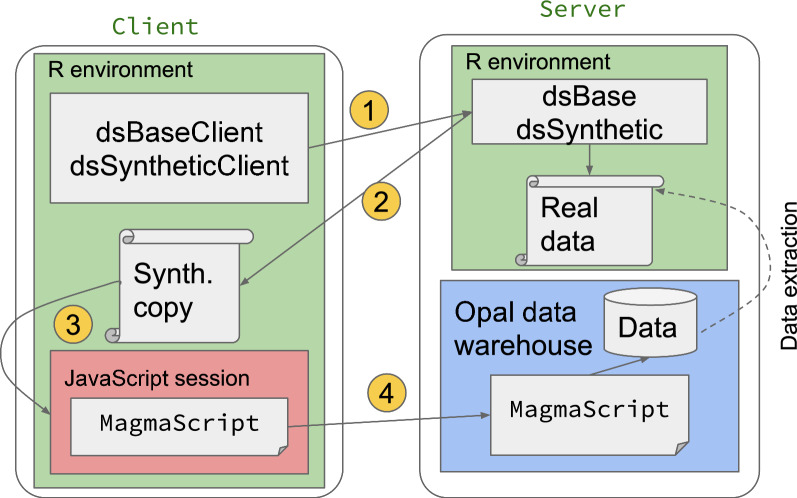
Central harmonisation via synthetic data without full access. The steps are the following: 1. User requests synthetic copy of real data 2. Synthetic copy generated on server side and passed to client 3. Synthetic data loaded into JavaScript session. User writes harmonisation code in (MagmaScript) on client side 4. MagmaScript code implemented on server side to run on real data

A schematic of this workflow is shown in Fig. 3.

In detail, the steps proposed are:

1. Generate synthetic data as described previously.
2. Start a JavaScript session on the client side.
3. Load the synthetic data into the session.
4. Write and test MagmaScript code in the session using synthetic data.
5. When testing is complete, copy the code into the Opal server to generate the harmonised version of the real data.

A worked example of this is detailed in the tutorial.

We note that remote, centralised harmonisation as described here could also be prototyped using DSLite and writing DataSHIELD, rather than MagmaScript, code. An example of this workflow would be harmonising to the Observational Medical Outcomes Partnership (OMOP) Common Data Model (as has been done in [12]).

### Minimising risk of disclosure

A concern with generating a synthetic dataset is that it might reveal too much information about the real data. In the case of using *synthpop*, existing work has assessed this risk to be low [13, 14].

We have also disabled built-in features of *synthpop* where certain options allow the return of real data (for example, setting the min.bucket value to 3 to prevent sampling from leaves that have data for very few individuals). When generating synthetic data via *simstudy*, this relies on non-disclosive results such as mean and standard deviation that can already be obtained via DataSHIELD, therefore there is no additional risk of disclosure.

### Discussion

We introduce a package (*dsSynthetic*) which provides functionality for generating synthetic data in DataSHIELD.

Synthetic data generation is also available as part of other privacy preserving frameworks like OpenSAFELY [15] and other packages [16–18]. A version of *synthpop* for DataSHIELD is offered in [16] but the *simstudy* method is not available, nor are the detailed workflows for generating synthetic data. Our package is simpler to use, offers different options to generate synthetic data (using packages like *simstudy* and *synthpop*) and comprehensive workflows for writing DataSHIELD scripts and data harmonisation

Determining privacy risks in synthetic data derived from real world healthcare data [19, 20] is also a fruitful area of research for future work. Indicators of the resemblance, utility and privacy of synthetic data will also be helpful [21–23].

## Limitations

We inherit the limitations of the underlying packages *simstudy* and *synthpop*. In particular, the user should exercise judgment about the number of variables requested in a synthetic dataset, so as to not overwhelm the server performing the generation.

## Data Availability

No data was generated in this study. All code and a tutorial are available here as an R bookdown format with executable code: https://tombisho.github.io/synthetic_bookdown All code is available from: https://github.com/tombisho/dsSynthetichttps://github.com/tombisho/dsSyntheticClient DataSHIELD is built on R and can be deployed using prebuilt docker images for easy installation. Our bookdown also documents a tutorial which can be followed with minimal client side package installations.

## Abbreviations

DSLite: DataSHIELD Lite
